# Supplementary material for: COVID-19 self-testing using antigen rapid diagnostic tests: Feasibility evaluation among health-care workers and general population in Malawi
Source: PLoS One. 2023 Jul 28;18(7):e0289291. doi: 10.1371/journal.pone.0289291 (PMC10381081; doi:10.1371/journal.pone.0289291)
Supplement: S1 Text — (DOCX) [file pone.0289291.s001.docx]

**Supplemental Material Caption**

This document contains supplemental material. It contains checklists as follows:

1. Table A. Checklist for Self-sampling- Standard Q
2. Table B. Checklist for Self-sampling- PanBio
3. Table C. Checklist for Self-testing- Standard Q
4. Table D. Checklist for Self-testing- PanBio

# **Table A. Checklist for Self-sampling- Standard Q**

| Task | Yes | No |
| --- | --- | --- |
| *Instruction 1*  Did participant wash or sanitise his/her hands? |  |  |
| *Instruction 2*  Did participant take each of the kit components out of the bag? |  |  |
| *Instruction 3 first part*  Was the seal of the solution tube opened correctly by peeling off? |  |  |
| *Instruction 3 second part*  Did the participant place the solution tube on a flat surface? |  |  |
| *Instruction 4*  Did the participant open the sterile swab correctly by peeling the paper edges apart from the top? |  |  |
| *Instruction 5 first part*  Did participant insert the swab into the first nostril to the correct depth (about 1.5cm)? |  |  |
| *Instruction 5 second part*  Did the participant rotate the swab 10 times in the first nostril? |  |  |
| *Instruction 5 third part*  Did the participant insert the same swab into the second nostril to the correct depth (about 1.5cm)? |  |  |
| *Instruction 5 fourth part*  Did the participant rotate the swab 10 times in the second nostril? |  |  |
| *Instruction 6 first part*  Did the participant insert the swab into the solution tube? |  |  |
| *Instruction 6 second part*  Did the participant stir the swab more than 10 times while squeezing the sides of the solution tube? |  |  |
| *Instruction 7 first part*  Did the participant remove the swab slowly while squeezing the sides of the tube to extract the liquid from the swab? |  |  |
| *Instruction 7 second part*  Did the participant place the swab in the plastic bag which had kits contents and dispose it in the bin? |  |  |
| *Instruction 7 third part*  Did the participant press the nozzle cap tightly onto the tube? |  |  |

# **Table B. Checklist for Self-sampling- Panbio**

| Task | Yes | No |
| --- | --- | --- |
| *Before starting*  Did participant wash or sanitise his/her hands? |  |  |
| *Instruction 2*  Did participant open the ziplock bag and take out all kit contents? |  |  |
| *Instruction 3*  Was buffer bottle opened correctly? |  |  |
| *Instruction 4*  Did participant squeeze correct amount of liquid with respect to the fill line? |  |  |
| *Instruction 5*  Did participant place the tube in the tray? |  |  |
| *Instruction 6*  Did participant open the swab protective package correctly at the stick end? |  |  |
| *Instruction 7 first part*  Did the participant insert the swab to the correct depth (about 2cm) in the first nostril? |  |  |
| *Instruction 7 second part*  Did the participant rotate the swab in the first nostril at least five times? |  |  |
| *Instruction 8 first part*  Did the participant insert the swab to the correct depth (about 2cm) in the second nostril? |  |  |
| *Instruction 8 second part*  Did the participant rotate the swab in the second nostril at least five times? |  |  |
| *Instruction 9 first part*  Did the participant insert the swab in the tube? |  |  |
| *Instruction 9 second part*  Did the participant swirl the swab in the tube at least five times? |  |  |
| *Instruction 9 third part*  Did the participant lift the soft end of the swab slightly above the fluid and squeeze the swab tip through the tube to remove remaining fluid? |  |  |
| *Instruction 10*  Did the participant break the swab handle at the break line and leave the swab in the tube? |  |  |
| *Instruction 11 first part*  Did the participant secure the blue cap on the top of the tube? |  |  |
| *Instruction 11 second part*  Did the participant place the tube on the tray? |  |  |

# **Table C. Checklist for Self-testing- Standard Q**

| **Steps** | **Observation** | | **Comment** |
| --- | --- | --- | --- |
| 1.Did participant wash or sanitise his/her hands? | ❑YES | ❑NO |  |
| 2.Did participant open the box and take out all box contents before testing? | ❑YES | ❑NO |  |
| 3.Did the participant check the expiry date at the back of the foil pouch? | ❑YES | ❑NO |  |
| 4.Did the participant Open the foil pouch and remove the test device and desiccant pack from the foil pouch? | ❑YES | ❑NO |  |
| 5.Did the participant open the Solution tube & Nozzle cap pouch tube correctly? | ❑YES | ❑NO |  |
| 6.Did the participant peel the seal of solution tube correctly? | ❑YES | ❑NO |  |
| 7.Did the participant set the solution tube on the stand hole of the package box correctly? | ❑YES | ❑NO |  |
| 8.Did the participant peel the sterile swab pouch correctly? Not holding the swab tip | ❑YES | ❑NO |  |
| 9.Did participant insert the swab into the left nostril to the correct depth (about 1.5cm)? | ❑YES | ❑NO |  |
| 10.Did participant insert the swab into the right nostril to the correct depth (about 1.5cm)? | ❑YES | ❑NO |  |
| 11.Did the participant rotate the swab 10 times in the left nostril? | ❑YES | ❑NO |  |
| 12.Did the participant rotate the swab 10 times in the right nostril? | ❑YES | ❑NO |  |
| 13.Did the participant insert the swab into the solution tube correctly? | ❑YES | ❑NO |  |
| 14.Did they squeeze the solution tube and stir the swab 10 times? | ❑YES | ❑NO |  |
| 15.Did the participant remove the swab while squeezing the sides of the tube? | ❑YES | ❑NO |  |
| 16.Did the participant press the nozzle cap tightly onto the tube? | ❑YES | ❑NO |  |
| 17.Did the participant apply 4 drops of extracted sample to the sample well of the test device? | ❑YES | ❑NO |  |
| 18.Did the participant read the test result in 15 minutes? | ❑YES | ❑NO |  |
| 19.Did the participant interpret the test result correctly? | ❑YES | ❑NO |  |

# **Table D. Checklist for Self-testing- Panbio**

| **Steps** | | | **Observation** | | **Comment** |
| --- | --- | --- | --- | --- | --- |
| Did participant wash or sanitise his/her hands? | | | ❑YES | ❑NO |  |
| Instruction 1  Did the participant check the expiry date on the box? | | | ❑YES | ❑NO |  |
| Instruction 2  Did participant open the box carefully without opening the individual components? | | | ❑YES | ❑NO |  |
| Instruction 3  Did the participant open the buffer bottle correctly (with the bottle upright)? | | | ❑YES | ❑NO |  |
| Instruction 4  Did the participant squeeze all the liquid from the buffer bottle into the tube? | | | ❑YES | ❑NO |  |
| Instruction 5  Did the participant place the tube on the kit box tray holder correctly? | | | ❑YES | ❑NO |  |
| Instruction 6  Did the participant open the swab protective package correctly? (Not holding the swab tip) | | | ❑YES | ❑NO |  |
| Instruction 7a  Did participant insert the swab into the left nostril to the correct depth (about 2 cm)? | | | ❑YES | ❑NO |  |
| Instruction 7b  Did the participant rotate the swab 5 times in the left nostril? | | | ❑YES | ❑NO |  |
| Instruction 8a  Did participant insert the swab into the right nostril to the correct depth (about 2cm)? | | | ❑YES | ❑NO |  |
| Instruction 8b  Did the participant rotate the swab 5 times in the right nostril? | | | ❑YES | ❑NO |  |
| Instruction 9a  Did the participant insert the swab into the solution tube correctly? | | | ❑YES | ❑NO |  |
| Instruction 9b  Did the participant swirl in the fluid 5 times while pushing against the wall of the Tube | | | ❑YES | ❑NO |  |
| Instruction 9c  Did the participant pinch the swab tip through the Tube to remove any remaining fluid? | | | ❑YES | ❑NO |  |
| Instruction 10  Did the participant snap the swab handle at the break line correctly? | | | ❑YES | ❑NO |  |
| Instruction 11  Did the participant press the nozzle cap tightly onto the tube and returned the Tube to the Tube Rack before proceeding to the next step? | | | ❑YES | ❑NO |  |
| Instruction 12  Did the participant tear the protective package and remove the Test Device and placed it on a flat surface? | | | ❑YES | ❑NO |  |
| Instruction 13a  Did the participant check or wait for bubbles to disappear? | | | ❑YES | ❑NO |  |
| Instruction 13b  Did the participant keep Tube vertical with the white cap pointed down? | | | ❑YES | ❑NO |  |
| **Steps** | | | **Observation** | | **Comment** |
| Instruction 14  Did the participant squeeze 5 drops of liquid from the Tube into the well on the Test Device? | | |  |  |  |
| Instruction 15  Did the participant read the test result in 15 minutes? | | | ❑YES | ❑NO |  |
| Did the participant interpret the test result correctly? | | | ❑YES | ❑NO |  |
| **Test results** |  | Test result from **study participant** sample?  ❑negative ❑positive ❑invalid | | | |
|  |  | Test result from **study staff sample**?  ❑negative ❑positive ❑invalid | | | |
