# Supplementary material for: COVID-19 self-testing using antigen rapid diagnostic tests: Feasibility evaluation among health-care workers and general population in Malawi
Source: PLoS One. 2023 Jul 28;18(7):e0289291. doi: 10.1371/journal.pone.0289291 (PMC10381081; doi:10.1371/journal.pone.0289291)
Supplement: S1 Table — (DOCX) [file pone.0289291.s002.docx]

# **S5 Table. Interpreting contrived panel results**

| **Question** | **Standard Q (N=333)** | | **PanBio (N=333)** | |
| --- | --- | --- | --- | --- |
|  | **No** | **Yes** | **No** | **Yes** |
| Was the participant's reading of positive dummy test correct? | 5 ( 1.5) | 329 (98.5) | 7 ( 2.1) | 325 (97.9) |
| Was the participant's reading of negative dummy test correct? | 2 ( 0.6) | 332 (99.4) | 3 ( 0.9) | 327 (99.1) |
| Was the participant's reading of invalid dummy test correct? | 11 ( 3.3) | 324 (96.7) | 13 ( 3.9) | 317 (96.1) |
