# Supplementary material for: COVID-19 self-testing using antigen rapid diagnostic tests: Feasibility evaluation among health-care workers and general population in Malawi
Source: PLoS One. 2023 Jul 28;18(7):e0289291. doi: 10.1371/journal.pone.0289291 (PMC10381081; doi:10.1371/journal.pone.0289291)
Supplement: S2 Table — (DOCX) [file pone.0289291.s003.docx]

**S3 Table. User views on self-sampling and self-testing**

|  | **Characteristic** | **Overall** | **Standard Q** | **Panbio** | **p-value*** |
| --- | --- | --- | --- | --- | --- |
| Number of participants | n | 663 | 332 | 331 |  |
| Recruitment site | QECH | 330 ( 50.2) | 167 ( 50.5) | 163 ( 49.8) | 0.938 |
|  | Lirangwe | 328 ( 49.8) | 164 ( 49.5) | 164 ( 50.2) |  |
| How hard was it for you to conduct the self-testing process correctly? | Not at all hard | 619 ( 93.4) | 301 ( 90.7) | 318 ( 96.1) | 0.013 |
|  | Somewhat hard | 22 ( 3.3) | 14 ( 4.2) | 8 ( 2.4) |  |
|  | Very hard | 22 ( 3.3) | 17 ( 5.1) | 5 ( 1.5) |  |
| How helpful was the demonstration that study staff did before you conducted self-testing? | Not at all helpful | 17 ( 2.6) | 4 ( 1.2) | 13 ( 3.9) | 0.085 |
|  | Somewhat helpful | 4 ( 0.6) | 2 ( 0.6) | 2 ( 0.6) |  |
|  | Very helpful | 642 ( 96.8) | 326 ( 98.2) | 316 ( 95.5) |  |
| Was any aspect of the demonstration unclear? | No | 661 ( 99.8) | 332 (100.0) | 329 ( 99.7) | 0.998 |
|  | Yes | 1 ( 0.2) | 0 ( 0.0) | 1 ( 0.3) |  |
| How helpful were the written instructions for self-testing? | Not at all helpful | 7 ( 1.1) | 2 ( 0.6) | 5 ( 1.5) | 0.117 |
|  | Somewhat helpful | 3 ( 0.5) | 3 ( 0.9) | 0 ( 0.0) |  |
|  | Very helpful | 653 ( 98.5) | 327 ( 98.5) | 326 ( 98.5) |  |
| Were there any written instructions which were not clear? | No | 661 ( 99.8) | 330 ( 99.7) | 331 (100.0) | 1.000 |
|  | Yes | 1 ( 0.2) | 1 ( 0.3) | 0 ( 0.0) |  |

^a^Chisquare test for categorical variables; t-test for continuous variables

QECH: Queen Elizabeth Central Hospital
