# Supplementary material for: COVID-19 self-testing using antigen rapid diagnostic tests: Feasibility evaluation among health-care workers and general population in Malawi
Source: PLoS One. 2023 Jul 28;18(7):e0289291. doi: 10.1371/journal.pone.0289291 (PMC10381081; doi:10.1371/journal.pone.0289291)
Supplement: Attachment — Submitted filename: PONE-D-22-25536 (1).pdf [file pone.0289291.s004.pdf]

PLOS ONE

COVID-19 self-testing using antigen rapid diagnostic tests: feasibility evaluation among health-care workers and general population in Malawi

--Manuscript Draft--

Manuscript Number:	PONE-D-22-25536
Article Type:	Research Article
Full Title:	COVID-19 self-testing using antigen rapid diagnostic tests: feasibility evaluation among health-care workers and general population in Malawi
Short Title:	COVID-19 self-testing
Corresponding Author:	Madalo Mukoka, MBBS, MSc Epidemiology<br>Kamuzu University of Health Sciences: University of Malawi College of Medicine<br>Blantyre, Malawi MALAWI
Keywords:	SARS-CoV-2; Self-testing; Covid-19; Self-sampling; Antigen Rapid Diagnostic Tests
Abstract:	Background : COVID-19 testing is critical for identifying cases to prevent transmission. SARS-CoV-2 self-testing has the potential to increase diagnostic testing capacity and to expand access to hard-to-reach areas in low-and-middle-income countries. We investigated the feasibility and acceptability of COVID-19 self-sampling and self-testing using SARS-CoV-2 Antigen-Rapid Diagnostic Tests (Ag-RDTs).<br>Methods : July 2021 to February 2022, we conducted a mixed-methods cross-sectional study examining self-sampling and self-testing using Standard Q and Panbio COVID-19 Ag Rapid Test Device in Urban and rural Blantyre, Malawi. Health care workers and adults (18y+) in the general population were systematically sampled.<br>Results: Overall, 1,330 participants were enrolled of whom 674 (56.0%) were female with 664 for self-sampling and 666 for self-testing. Mean age was 30.7y (standard deviation [SD] 9.6). Self-sampling usability threshold for Standard Q was 273/333 (82.0%: 95% CI 77.4% to 86.0%) and 261/331 (78.8%: 95% CI 74.1% to 83.1%) for Panbio. Self-testing threshold was 276/335 (82.4%: 95% CI 77.9% to 86.3%) and 300/332 (90.4%: 95% CI 86.7% to 93.3%) for Standard Q and Panbio, respectively. Agreement between self-sample results and professional test results was 325/325 (100%) and 322/322 (100%) for Standard Q and Panbio, respectively. For self-testing, agreement was 332/333 (99.7%: 95% CI 98.3 to 100%) for Standard Q and 330/330 (100%: 95% CI 99.8 to 100%) for Panbio. Odds of achieving self-sampling threshold increased if the participant was recruited from an urban site (odds ratio [OR] 2.15 95% CI 1.44 to 3.23, P < .01. Compared to participants with primary school education those with secondary and tertiary achieved higher self-testing threshold OR 1.88 (95% CI 1.17 to 3.01), P = .01 and 4.05 (95% CI 1.20 to13.63), P = .02, respectively.<br>Conclusions: One of the first studies to demonstrate high feasibility of self-testing using SARS-CoV-2 Ag-RDTs in low- and middle-income countries potentially supporting large scale-up.
Order of Authors:	Madalo Mukoka, MBBS, MSc Epidemiology
	Euphemia Sibanda, PhD
	Constancia Watadzaushe, BSc
	Moses Kumwenda, PhD
	Florence Abok, MA
	Elizabeth L Corbett, MD PhD
	Elena Ivanova, PhD
	Augustine Talumba Choko, PhD
Additional Information:	
Question	Response
Financial Disclosure	This research was funded by UNITAID (grant number KFW P09022-00) through the

Powered by Editorial Manager® and ProduXion Manager® from Aries Systems Corporation

Enter a financial disclosure statement that describes the sources of funding for the work included in this submission. Review the submission guidelines for detailed requirements. View published research articles from *PLOS ONE* for specific examples.<br><br>This statement is required for submission and **will appear in the published article** if the submission is accepted. Please make sure it is accurate.<br><br>**Unfunded studies**<br>Enter: *The author(s) received no specific funding for this work.*<br><br>**Funded studies**<br>Enter a statement with the following details:<br>• Initials of the authors who received each award<br>• Grant numbers awarded to each author<br>• The full name of each funder<br>• URL of each funder website<br>• Did the sponsors or funders play any role in the study design, data collection and analysis, decision to publish, or preparation of the manuscript?<br>• **NO** - Include this sentence at the end of your statement: *The funders had no role in study design, data collection and analysis, decision to publish, or preparation of the manuscript.*<br>• **YES** - Specify the role(s) played.<br><br>* typeset	Foundation For Innovative New Diagnostics. The funder had no role in the study design, data collection, analysis, decision to publish or preparation of the manuscript.
**Competing Interests**<br><br>Use the instructions below to enter a competing interest statement for this submission. On behalf of all authors, disclose any competing interests that could be perceived to bias this work—acknowledging all financial support and any other relevant financial or non-financial competing interests.<br><br>This statement is required for submission and **will appear in the published article** if	The authors have declared that no competing interests exist.

the submission is accepted. Please make sure it is accurate and that any funding sources listed in your Funding Information later in the submission form are also declared in your Financial Disclosure statement.<br><br>View published research articles from *PLOS ONE* for specific examples.<br><br>**NO authors have competing interests**<br><br>Enter: *The authors have declared that no competing interests exist.*<br><br>**Authors with competing interests**<br><br>Enter competing interest details beginning with this statement:<br><br>*I have read the journal's policy and the authors of this manuscript have the following competing interests: [insert competing interests here]*<br><br>* typeset	
**Ethics Statement**<br><br>Enter an ethics statement for this submission. This statement is required if the study involved:<br><br>• Human participants<br>• Human specimens or tissue<br>• Vertebrate animals or cephalopods<br>• Vertebrate embryos or tissues<br>• Field research<br><br>Write "N/A" if the submission does not require an ethics statement.<br><br>General guidance is provided below. Consult the submission guidelines for detailed instructions. **Make sure that all information entered here is included in the Methods section of the manuscript.**	The study was conducted according to the guidelines of the Declaration of Helsinki, and approved by the Malawi College of Medicine Research Ethics Committee of Kamuzu University of Health Sciences (Reg No: P.03/21/3277) and the World Health Organization Research Ethics Review Committee (Protocol ID: CERC.0104). Informed consent was obtained from all participants involved in the study.

**Format for specific study types**<br><br>**Human Subject Research (involving human participants and/or tissue)**<br>• Give the name of the institutional review board or ethics committee that approved the study<br>• Include the approval number and/or a statement indicating approval of this research<br>• Indicate the form of consent obtained (written/oral) or the reason that consent was not obtained (e.g. the data were analyzed anonymously)<br><br>**Animal Research (involving vertebrate animals, embryos or tissues)**<br>• Provide the name of the Institutional Animal Care and Use Committee (IACUC) or other relevant ethics board that reviewed the study protocol, and indicate whether they approved this research or granted a formal waiver of ethical approval<br>• Include an approval number if one was obtained<br>• If the study involved *non-human primates*, add *additional details* about animal welfare and steps taken to ameliorate suffering<br>• If anesthesia, euthanasia, or any kind of animal sacrifice is part of the study, include briefly which substances and/or methods were applied<br><br>**Field Research**<br><br>Include the following details if this study involves the collection of plant, animal, or other materials from a natural setting:<br>• Field permit number<br>• Name of the institution or relevant body that granted permission	
**Data Availability**<br><br>Authors are required to make all data underlying the findings described fully available, without restriction, and from the time of publication. PLOS allows rare exceptions to address legal and ethical concerns. See the PLOS Data Policy and FAQ for detailed information.	Yes - all data are fully available without restriction

A Data Availability Statement describing where the data can be found is required at submission. Your answers to this question constitute the Data Availability Statement and **will be published in the article**, if accepted.<br><br>**Important:** Stating 'data available on request from the author' is not sufficient. If your data are only available upon request, select 'No' for the first question and explain your exceptional situation in the text box.<br><br>Do the authors confirm that all data underlying the findings described in their manuscript are fully available without restriction?	
**Describe where the data may be found in full sentences. If you are copying our sample text, replace any instances of XXX with the appropriate details.**<br><br>• If the data are **held or will be held in a public repository**, include URLs, accession numbers or DOIs. If this information will only be available after acceptance, indicate this by ticking the box below. For example: *All XXX files are available from the XXX database (accession number(s) XXX, XXX.*).<br>• If the data are all contained **within the manuscript and/or Supporting Information files**, enter the following: *All relevant data are within the manuscript and its Supporting Information files.*<br>• If neither of these applies but you are able to provide **details of access elsewhere**, with or without limitations, please do so. For example:<br><br>*Data cannot be shared publicly because of [XXX]. Data are available from the XXX Institutional Data Access / Ethics Committee (contact via XXX) for researchers who meet the criteria for access to confidential data.*<br><br>*The data underlying the results presented in the study are available from (include the name of the third party*	The trial protocol and dataset supporting the conclusions of this article are available via the London School of Hygiene & Tropical Medicine Data Compass https://datacompass.lshtm.ac.uk/

and contact information or URL).<br>• This text is appropriate if the data are owned by a third party and authors do not have permission to share the data.<br><br>* typeset	
Additional data availability information:	

Powered by Editorial Manager® and ProduXion Manager® from Aries Systems Corporation

Manuscript

Click here to access/download;Manuscript;CV19_Self-testing_Plos One_final.docx

COVID-19 self-testing using antigen rapid diagnostic tests: feasibility evaluation among health-care workers and general population in Malawi

Short title: COVID-19 self-testing

Madalo Mukoka,[1,*] Euphemia Sibanda[2], Constancia Watadzaushe[2], Moses Kumwenda[1, 3], Florence Abok[4], Elizabeth L. Corbett[5], Elena Ivanova[4], Augustine Talumba Choko[3]

1. Department of Pathology, Helse-Nord Tuberculosis Initiative, Kamuzu University of Health Sciences, Blantyre, Malawi
2. The Centre for Sexual Health and HIV/AIDS Research Zimbabwe, Harare, Zimbabwe
3. Public Health and Policy group, Malawi Liverpool Wellcome Trust Clinical Research Programme, Blantyre, Malawi
4. Foundation for Innovative New Diagnostics, Geneva, Switzerland
5. Clinical Research Department, London School of Hygiene & Tropical Medicine, London, UK

***Correspondence to:**

Madalo Mukoka

E-mail: madalo.mukoka@gmail.com (MM)

Mobile phone: +265 (0) 888 518 464

Abstract

Background: COVID-19 testing is critical for identifying cases to prevent transmission. SARS-CoV-2 self-testing has the potential to increase diagnostic testing capacity and to expand access to hard-to-reach areas in low-and-middle-income countries. We investigated the feasibility and acceptability of COVID-19 self-sampling and self-testing using SARS-CoV-2 Antigen-Rapid Diagnostic Tests (Ag-RDTs).

Methods: July 2021 to February 2022, we conducted a mixed-methods cross-sectional study examining self-sampling and self-testing using Standard Q and Panbio COVID-19 Ag Rapid Test Device in Urban and rural Blantyre, Malawi. Health care workers and adults (18y+) in the general population were systematically sampled.

Results: Overall, 1,330 participants were enrolled of whom 674 (56.0%) were female with 664 for self-sampling and 666 for self-testing. Mean age was 30.7y (standard deviation [SD] 9.6). Self-sampling usability threshold for Standard Q was 273/333 (82.0%: 95% CI 77.4% to 86.0%) and 261/331 (78.8%: 95% CI 74.1% to 83.1%) for Panbio. Self-testing threshold was 276/335 (82.4%: 95% CI 77.9% to 86.3%) and 300/332 (90.4%: 95% CI 86.7% to 93.3%) for Standard Q and Panbio, respectively. Agreement between self-sample results and professional test results was 325/325 (100%) and 322/322 (100%) for Standard Q and Panbio, respectively. For self-testing, agreement was 332/333 (99.7%: 95% CI 98.3 to 100%) for Standard Q and 330/330 (100%: 95% CI 99.8 to 100%) for Panbio. Odds of achieving self-sampling threshold increased if the participant was recruited from an urban site (odds ratio [OR] 2.15 95% CI 1.44 to 3.23, $P < .01$. Compared to participants with primary school education those with secondary and tertiary achieved higher self-testing threshold OR 1.88 (95% CI 1.17 to 3.01), $P = .01$ and 4.05 (95% CI 1.20 to13.63), $P = .02$, respectively.

Conclusions: One of the first studies to demonstrate high feasibility of self-testing using SARS-CoV-2 Ag-RDTs in low- and middle-income countries potentially supporting large scale-up.

Introduction

Only around 0.2% of people in Africa had tested for severe acute respiratory syndrome coronavirus 2 (SARS-CoV-2) the infection that causes COVID-19 in August 2020.[1] By contrast, 19.5% of Americans had tested by the same time[1] since COVID-19 emergence in December 2019.[2, 3] These contrasting trends have continued to exist with widening unequal access to testing, treatment and vaccination between high income countries and low and middle income countries despite four or more global epidemic waves.[4] Testing remains the most critical step for identification and isolation of COVID-19 cases to prevent transmission. [5] In many resource-limited settings, demand for tests often exceeds supply[6], hence there is need to improve diagnostic capacity. SARS-CoV-2 rapid antigen tests (Ag-RDTs) are recommended to complement nucleic acid amplification tests (NAAT) for diagnosis[7], which in resource-limited settings are often hard to implement because they require specialised skills and limited centralized laboratory capacity, associated with long turnaround times, and high costs to both the health system and patients.

COVID-19 self-testing was strongly recommended by the World Health Organization (WHO) in March 2022 as an additional strategy to complement professionally administered testing services.[8] Self-testing, which has been successfully used in other disease areas such as HIV where uptake is ubiquitously high including among hard to reach and key populations.[9-11] In general, COVID-19 self-testing has the potential to increase diagnostic capacity for COVID-19 and reduce access barriers as well as prevailing inequalities due to ease of distribution and being extremely convenient.[12] However, COVID-19 self-testing has so far been widely implemented and made available in high income countries.[12-14] As with HIV self-testing, lack of linkage for next steps with COVID-19 is a potential concern due to stigma, loss of economic opportunities due to isolation implications, and fear of complications including death.

Being able to self-test rests on the assumption that individuals would be able to take their own sample (self-sampling).[15] However, in settings with low exposure to technology and the ability to correctly follow instructions such assumptions may be faulty.[16] Thus, early work including optimization of instructions for use through iterative cognitive interviews is essential to ensure correct use of self-tests.[16] Here we investigated the feasibility and acceptability of COVID-19 self-sampling and self-testing using SARS-CoV-2 Ag-rapid diagnostics tests (RDTs) in Malawi.

Materials and Methods

Study design

A mixed-methods cross-sectional study examining self-sampling and self-testing for COVID-19 using STANDARD Q COVID-19 Ag Test (SD Biosensor) and Panbio COVID-19 Ag Rapid Test Device (Abbott Rapid Diagnostics). We conducted the study under five components. These are: cognitive interviews to refine instructions for use (IFUs) for self-sampling, observational cross-sectional study of self-sampling, cognitive interviews to refine instructions for use for self-testing, observational cross-sectional study of self-testing, and in-depth interviews (IDIs) to understand participant views on self-sampling and self-testing.

Setting

Recruitment was conducted between July 2021 to February 2022 from Lirangwe Primary Health Centre from rural Blantyre and from Queen Elizabeth Central Hospital (QECH) from urban Blantyre, Malawi.

Participants

We recruited health care workers and members of the general public from the recruitment sites. To be eligible, participants needed to be 18 years or older, feeling well enough to comfortably conduct study activities, not having recent history of excessive nose bleeds, and having given consent. All health workers from the two health facilities were offered the choice to participate in the study with exclusion only done if ineligible. An additional eligibility criteria which was later relaxed due to scarcity of participants with waning wave concerned individuals being on the list to be tested for COVID-19 by the national systems. General public participants were systematically sampled from outpatient departments. An anterior nasal swab for COVID-19 was done for both self-sampling and self-testing following a short in-person demonstration by a member of staff.

Participants were observed in-person during self-sampling and self-testing, and a checklist (S1-S4 appendix) was completed to document whether each task was done correctly. A trained researcher then tested the collected sample using a COVID-19 Ag RDT during the self-sampling component of the study. Participants tested their own collected sample during the self-testing component of the study. The trained researcher collected and tested an anterior nasal confirmatory sample using an Ag RDT during both the self-sampling and self-testing components.

Variables

For the cognitive interviews, the main output was to have refined IFUs in the local language (Chichewa) and in English. The first primary outcome was the percentage of participants who attained a usability threshold for self-sampling, defined as correct execution of all critical

instructions during the self-sampling process for each kit. Correct self-sampling was referred to as self-sampling accuracy. The second primary outcome was the percentage of participants who attained a usability threshold for self-testing, defined as correct execution of all critical instructions during the self-testing process. Correct self-testing was referred to as self-testing accuracy. User views regarding self-sampling and self-testing were the main outcomes from the IDIs. Potential confounders for accuracy were age, sex, literacy and prior exposure to COVID-19 testing.

Data sources/ measurement

Qualitative data from cognitive interviews and IDIs were tape recorded before being translated and transcribed. Pre- and post-test questionnaires were administered in-person using Open Data Kit (ODK) loaded on tablets. A checklist (S1-S4 appendix) was completed by a member of staff to document whether each instruction was done correctly as a measure of accuracy. Results obtained by a trained researcher from testing the collected self-sample and a sample collected by the researcher were recorded on the checklist. For self-testing accuracy, participant's self-test self-read results were compared to RDT sampling and testing conducted by the researcher. Participants' reading of pre-made cassettes of negative, positive and invalid results was also recorded.

Bias

The main source of bias is in the assessment by the research staff using a checklist of the performance of the participant on the IFU. A staff member who was more punitive may have harshly rated performance as incorrect while a more forgiving one may have rated performance differently. However, the fact that more than seven staff members were involved in the rating may have minimized such bias.

Study size

We aimed to recruit and purposively sample 120 participants for cognitive interviews for self-sampling and self-testing for both test kits. For self-sampling and self-testing, we conservatively assumed that 70% to 80% of participants will be able to correctly follow instructions and self-sample or self-test for COVID-19. For the sample proportion to be estimated to within +/-0.05 (5%) using the 95% confidence level, a sample of 323 participants were required. Thus, a total of 1,320 participants were needed: 330 per test kit for self-sampling and self-testing. A purposive sample of 120 participants was needed for the IDIs: 60 self-sampling and 60 self-testing participants.

Quantitative variables

A binary variable was generated for the first and secondary primary outcomes of achieving the threshold (accuracy) for either self-sampling or self-testing. This was coded as 1 for participants with a maximum score on the critical steps based on the checklist and 0 otherwise. Test result variables were coded as 1 for positive and 0 for negative.

Statistical methods

Analysis used R[17] with 0.05 as an indicator of statistical significance. Frequencies were computed for categorical variables while mean and standard deviation (SD) or median and (inter quartile range) were computed for continuous variables that were normally distributed or skewed, respectively. We computed the proportion achieving accuracy along with Binomial Exact confidence intervals (CIs) for self-sampling and self-testing for each test kit. Similarly, we computed the proportion of self-test results that agreed with staff conducted RDT test results

along with Binomial Exact confidence intervals (CIs) for each test kit. Logistic regression was used to examine factors associated with accuracy.

Ethics Statement

The study was conducted according to the guidelines of the Declaration of Helsinki, and approved by the Malawi College of Medicine Research Ethics Committee of Kamuzu University of Health Sciences (Reg No: P.03/21/3277) and the World Health Organization Research Ethics Review Committee (Protocol ID: CERC.0104). Informed consent was obtained from all participants involved in the study.

Results

Participants and Descriptive data

A total of 120 participants were recruited for self-sampling and self-testing cognitive interviews. Of 723 screened for eligibility 664 (91.8%) were recruited for self-sampling with mean age of 31.4y (standard deviation [SD]: 9.8) and 357/664 (53.8%) were male (Table 1). For self-testing, 666 (95.4%) were recruited of 698 screened for eligibility; mean age was 30.6y (standard deviation [SD]: 9.6) with 293/666 (44.0%) being male (Table 2). The main exclusion was being under 18 years. Sixty participants were recruited for IDIs.

Outcome data

The cognitive interviews showed that participants in both rural and urban communities were able to follow the IFUs with no major suggestions for changes. Notable changes to IFUs included: making introductory text stand out to catch attention, enhancing clarity of IFUs such as by expanding text, adding labels on images, selecting words or phrases that could be well understood locally. Insertion of test swab to correct depth (1.5cm or 2cm) was illustrated by reference to inserting up to thumbnail depth.

Table 1. Baseline Characteristics: Self-sampling

Variable	Characteristic	Overall	Standard Q	Panbio	p-value[a]
Number of participants	n	664	331	333	
Sex	Male	357 (53.8)	176 (53.2)	181 (54.4)	0.820
	Female	307 (46.2)	155 (46.8)	152 (45.6)	
Age (years)	mean (SD)	31.4 (9.8)	31.8 (10.3)	31.0 (9.3)	0.313
Ever tested for COVID-19?	No	568 (87.0)	303 (91.8)	265 (82.0)	<0.001
	Yes	85 (13.0)	27 (8.2)	58 (18.0)	
Marital status	Divorced	37 (5.7)	20 (6.1)	17 (5.3)	0.512
	Separated	33 (5.1)	15 (4.5)	18 (5.6)	
	Widowed	14 (2.1)	8 (2.4)	6 (1.9)	
	Never married	180 (27.6)	82 (24.8)	98 (30.3)	
	Married	389 (59.6)	205 (62.1)	184 (57.0)	
Money earned per month (MWK)	mean (SD)	68191 (96060)	57471 (90035)	79111 (100803)	0.004
Able to read a newspaper?	No	42 (6.4)	29 (8.8)	13 (4.0)	0.020
	Yes	610 (93.6)	300 (91.2)	310 (96.0)	
Highest level of formal schooling	Never been to school	25 (3.9)	18 (5.5)	7 (2.2)	<0.001
	Primary	165 (25.4)	101 (31.0)	64 (19.8)	
	Secondary no MSCE	216 (33.3)	118 (36.2)	98 (30.3)	
	Secondary with MSCE	131 (20.2)	54 (16.6)	77 (23.8)	
	Tertiary	112 (17.3)	35 (10.7)	77 (23.8)	
Number of people in household	mean (SD)	4.2 (1.8)	4.2 (1.8)	4.2 (1.8)	0.903
Number of rooms in household	mean (SD)	2.5 (1.0)	2.4 (1.0)	2.6 (1.1)	0.032
Number of households per dwelling	mean (SD)	1.5 (1.3)	1.3 (0.8)	1.7 (1.7)	<0.001
Enough food / essentials for 14 days?	No	412 (63.1)	215 (65.2)	197 (61.0)	0.307
	Yes	241 (36.9)	115 (34.8)	126 (39.0)	
Recruitment site	QECH	331 (49.8)	165 (49.8)	166 (49.8)	1.000
	Lirangwe	333 (50.2)	166 (50.2)	167 (50.2)	

[a]Chisquare test for categorical variables; t-test for continuous variables

SD: standard deviation; QECH: Queen Elizabeth Central Hospital

Table 2. Baseline Characteristics: Self-testing

Variable	Characteristic	Overall	Standard Q	Panbio	p-value[a]
Number of participants	n	664	336	328	
Sex	Male	292 (44.0)	138 (41.1)	154 (47.0)	0.148
	Female	372 (56.0)	198 (58.9)	174 (53.0)	
Age (years)	mean (SD)	30.7 (9.6)	30.8 (9.8)	30.52 (9.3)	0.724
Ever tested for COVID-19?	No	603 (91.5)	303 (91.0)	300 (92.0)	0.659
	Yes	56 (8.5)	30 (9.0)	26 (8.0)	
Marital status	Divorced	37 (5.6)	15 (4.5)	22 (6.8)	0.208
	Separated	47 (7.2)	24 (7.2)	23 (7.1)	
	Widowed	25 (3.8)	13 (3.9)	12 (3.7)	
	Never married	195 (29.7)	88 (26.5)	107 (32.9)	
	Married	353 (53.7)	192 (57.8)	161 (49.5)	
Money earned per month (MWK)	mean (SD)	67802 (123668)	70359 (153192)	65190 (83531)	0.591
Able to read a newspaper?	No	59 (8.9)	29 (8.7)	30 (9.2)	0.922
	Yes	601 (91.1)	305 (91.3)	296 (90.8)	
Highest level of formal schooling	Never been to school	33 (5.0)	19 (5.7)	14 (4.3)	0.209
	Primary	208 (31.5)	109 (32.6)	99 (30.4)	
	Secondary no MSCE	220 (33.3)	117 (35.0)	103 (31.6)	
	Secondary with MSCE	143 (21.7)	68 (20.4)	75 (23.0)	
	Tertiary	56 (8.5)	21 (6.3)	35 (10.7)	
Number of people in household	mean (SD)	4.2 (1.6)	4.2 (1.7)	4.3 (1.6)	0.548
Number of rooms in household	mean (SD)	2.5 (1.2)	2.5 (1.0)	2.5 (1.4)	0.559
Number of households per dwelling	mean (SD)	1.5 (1.3)	1.5 (1.6)	1.5 (0.9)	0.516
Enough food / essentials for 14 days?	No	351 (53.3)	188 (56.5)	163 (50.0)	0.113
	Yes	308 (46.7)	145 (43.5)	163 (50.0)	
Recruitment site	QECH	334 (50.8)	169 (51.1)	165 (50.6)	0.971
	Lirangwe	323 (49.2)	162 (48.9)	161 (49.4)	

Main results

Self-sampling accuracy was 273/333 (82.0%: 95% CI: 77.4 to 86.0) for Standard Q and 261/331 (78.8: 95% CI: 74.1% to 83.1%) for Panbio (Table 3). The percentage agreement between the test results from the participant and the study staff was 100% for both kits in Malawi (Table 3).

Self-testing accuracy was 276/335 (82.4%: 95% CI: 77.9 to 86.3) for Standard Q and 300/332 (90.4%: 95% CI: 86.7 to 93.3) for Panbio (Table 3). The percentage agreement between the test results from the participant and the study staff was 99.7% (95% CI: 98.3-100%) for Standard Q with only one false negative self-test self-read result.

Table 3. Self-sampling and Self-testing Accuracy

	**Standard Q**						**Panbio**			
	**N**	**n**	**%**	**95% CI**		**N**	**n**	**%**	**95% CI**	
Met self-sampling threshold[a]	333	273	82.0	77.4	86.0	331	261	78.8	74	83.1
Met self-testing threshold	335	276	82.4	77.9	86.3	332	300	90.4	86.7	93.3
Agreement with professional test										
Self-sampling	322	322	100	99	100	325	325	100	100	100
Self-testing	333	332	99.7	98	100	330	330	100	100	100

[a]Threshold: participant performing all critical steps correctly

CI: confidence interval

Up to 95% of the critical steps were performed correctly on either test kit for both self-sampling and self-testing (Table 4).

Table 4. User errors for Standard Q and PanBio kits

	**Standard Q (N = 331)**		**Panbio (N = 331)**	
	**Yes**	**No**	**Yes**	**No**
Did the participant place the tube on the kit box tray holder or flat surface correctly?	327 (97.6)	8 (2.4)	329 (99.4)	2 (0.6)
Did participant insert the swab into the left nostril to the correct depth (about 1.5cm or 2cm)?	327 (97.6)	8 (2.4)	332 (100)	0 (0.0)
Did the particiq234pant rotate the swab 5 or 10 times in the left nostril?	325 (97.0)	10 (3.0)	327 (98.5)	5 (1.5)
Did participant insert the swab into the right nostril to the correct depth (about 1.5cm or 2cm)?	327 (98.5)	5 (1.5)	329 (99.4)	2 (0.6)
Did the participant rotate the swab 5 or 10 times in the right nostril?	324 (97.0)	10 (3.0)	330 (99.4)	2 (0.6)
Did the participant insert the swab into the solution tube correctly?	331 (98.8)	4 (1.2)	328 (99.4)	2 (0.6)
Did the participant swirl in the fluid 5 or 10 times while pushing against the wall of the tube?	325 (97.3)	9 (2.7)	323 (97.6)	8 (2.4)
Did the participant remove the swab slowly while squeezing the sides of the tube to extract the liquid from the swab?	310 (92.5)	25 (7.5)	316 (95.5)	15 (4.5)
Did the participant press the nozzle cap tightly the tube?	326 (97.9)	7 (2.1)	330 (99.7)	1 (0.3)
Did the participant squeeze 4 or 5 drops of liquid from the tube into the well on the test device?	319 (95.5)	15 (4.5)	330 (99.7)	1 (0.3)
Did the participant read the test result in 15 minutes?	333 (99.7)	1 (0.3)	325 (97.9)	7 (2.1)
Did the participant interpret the test result correctly?	328 (98.5	2 (1.5)	329 (99.7)	1 (0.3)

Standard Q: swab 10 times, depth 2cm, 4 drops

The odds of self-sampling accuracy increased 2-fold for participants from QECH compared to participants from Lirangwe primary health centre odds ratio (OR) 2.15 (95% CI 1.44 to 3.23, $P <$ 0.1 (Table 5). There appeared to be a linear trend towards increased odds of attaining self-testing accuracy with increasing levels of education, P for trend 0.01.

Table 5. Factors associated with self-sampling and self-testing accuracy

		Self-sampling (N = 641)				Self-testing (N = 637)			
		Unadjusted				Unadjusted			
**Variable**	**Characteristic**	**OR**	**95% CI**		**p-value**	**OR**	**95% CI**		**p-value**
Age	Yearly increase	0.99	0.97	1.01	0.247	0.99	0.97	1.01	0.299
Sex	Female	1.00				1.00			
	Male	1.00	0.68	1.48	0.991	0.84	0.54	1.33	0.459
Site	Lirangwe	1.00				1.00			
	QECH	2.15	1.44	3.23	<0.001	1.47	0.93	2.32	0.097
Literacy	No	1.00				1.00			
	Yes	0.57	0.22	1.49	0.251	1.21	0.57	2.55	0.625
Highest level of education attained?	Primary school	1.00				1.00			
	Never been school	2.75	0.61	12.3	0.186	1.67	0.55	5.02	0.362
	Secondary	0.91	0.58	1.44	0.686	1.88	1.17	3.01	0.009
	Tertiary	2.09	1.03	4.25	0.041	4.05	1.20	13.63	0.024
Marital Status	Divorced/separated/widowed	1.00				1.00			
	Never married	2.10	1.06	4.14	0.033	2.41	1.26	4.61	0.008
	Married	1.07	0.61	1.89	0.820	1.89	1.09	3.28	0.023
Ever tested for COVID-19?	No	1.00				1.00			
	Yes	1.04	0.58	1.86	0.905	1.25	0.52	3.02	0.619

OR: odds ratio; CI: confidence interval; QECH: Queen Elizabeth Central Hospital

Other analyses

Nearly all in-depth interview participants reported that self-testing was highly acceptable because it was convenient, empowering and private.

Most participants had no problems interpreting contrived panel results with 99% correctly interpreting positive and negative results correctly although 96% correctly interpreted invalid results on either test kit (S5 Table). Up to 90.7% Standard Q and 96.1% Panbio participants found instructions “not at all hard” when asked on exit interviews (S6 Table).

Discussion

Key results

This is one of first studies to be conducted on COVID-19 self-testing in low- and middle-income countries and generally indicates that participants in both rural and urban communities in Malawi can self-test correctly for COVID-19. The results of this study show that 82% and 90% of participants were able to self-test for COVID-19 with no supervision following a brief demonstration using Standard Q and PanBio test kits, respectively. Of further note, all self-test results agreed 100% with professionally conducted RDTs for PanBio kit whereas agreement was 99.7% for Standard Q. Similarly, 82% of participants were able to correctly self-sample for COVID-19 using Standard Q compared to 79% using PanBio. COVID-19 self-testing was rated as highly acceptable during in-depth interviews.

Current strategies for COVID-19 testing in high income countries are largely dependent on Ag-RDT self-sampling and self-testing[18-20] with over-the-counter self-test kits available for purchase in a wide range of countries.[14, 21] The limited data available in resource-poor settings suggest that, as with HIV self-testing, diagnostic accuracy is not as great with untrained lay users as with trained professionals, mainly affecting sensitivity.[13, 18, 22] Our results on the other hand show that self-testing accuracy improved markedly with a short demonstration supporting previous findings observed with HIV self-testing.[10, 23] Our study investigated self-testing with two kits that were already approved for use in Malawi. However, there are numerous Ag-RDT tests packaged for COVID-19 self-testing that have met performance standards and been approved by Regulatory Authorities such as the FDA, that may yield similar promising results.[24, 25]

Limitations

There are notable limitations with our study. Firstly, there was a small number of positive self-test results. Although this does not affect the reading of correct results and indeed completing critical steps correctly as assessed here it may be important as it is likely to affect sensitivity.[26] Reassuringly, up to 99% of participants correctly interpreted contrived positive results on either kit. Secondly, there was potential for assessment bias resulting from subjective judgement on the checklist used by research staff for assessing performance of the participant on each instruction. The impact of this bias could be bi-directional depending on whether the staff was harsh – leading to poor rating, or more lenient resulting in more participants being passed as correctly following instructions.

Generalisability

This study demonstrates high acceptability and feasibility of COVID-19 self-testing.[8] The findings are very similar to results reported in other self-testing areas including HIV[27] and hepatitis C virus (HCV)[28]. Thus, we posit that the findings are generalizable to many resource settings and populations including those with limited literacy. However, some support may be useful for specific settings and users – such as older age groups and those with lower literacy. Lessons learned from introduction and scale-up of other self-testing approaches such as HIV and HCV may be appliable here to accelerate adaptation plans and efforts in LMIC.

Conclusions

This is one the first studies to demonstrate high usability and acceptability self-testing using SARS-CoV-2 Ag-RDTs among both general and health-care worker populations in low- and middle-income countries. While most users collected their own samples and self-tested with ease, participants noted demonstrations were helpful and could be important in some settings and

populations, such as older age groups and those with low literacy levels. COVID-19 self-testing is an important strategy for further consideration as it may be a promising tool for increasing access to and uptake of COVID-19 testing services as well as strategies to reduce transmission and linkage to further care, treatment and support services.

Acknowledgements

The authors would like to thank all study participants, participating health facilities, and project team members for their contributions to the successful completion of this project.

Authors' contributions

Conceptualization, MM, ES, CW, MK, FA, ELC, EI and ATC; Data curation, MM and AC; Formal analysis, MM, ES, MK and AC; Funding acquisition, ES, FA, EC, EI and AC; Investigation, MM, ES, CW, MK, FA and AC; Methodology, MM, ES, CW, MK, FA and AC; Project administration, MM, ES, CW, MK, FA, EC, EI and AC; Resources, MM, EI and AC; Supervision, ES, MK, FA, EC, EI and AC; Validation, MM, ES, EC, EI and AC; Visualization, ES and AC; Writing – original draft, MM and AC; Writing – review & editing, ES, CW, MK, FA, EC and EI.

Data Availability statement

The trial protocol and dataset supporting the conclusions of this article are available via the London School of Hygiene & Tropical Medicine Data Compass https://datacompass.lshtm.ac.uk/

References

1. Chitungo I, Dzobo M, Hlongwa M, Dzinamarira T. COVID-19: Unpacking the low number of cases in Africa. Public health in practice (Oxford, England). 2020;1:100038. Epub 2021/06/27. doi: 10.1016/j.puhip.2020.100038. PubMed PMID: 34173573; PubMed Central PMCID: PMCPMC7485446.

2. World Health Organization. WHO-convened global study of origins of SARS-CoV-2: China Part. Joint WHO-China Study Team report: 14 January-10 February 2021. Geneva, Switzerland: WHO, 2021.

3. Zoumpourlis V, Goulielmaki M, Rizos E, Baliou S, Spandidos DA. [Comment] The COVID‑19 pandemic as a scientific and social challenge in the 21st century. Molecular medicine reports. 2020;22(4):3035-48. Epub 2020/09/19. doi: 10.3892/mmr.2020.11393. PubMed PMID: 32945405; PubMed Central PMCID: PMCPMC7453598.

4. Zhang SX, Arroyo Marioli F, Gao R, Wang S. A Second Wave? What Do People Mean by COVID Waves? - A Working Definition of Epidemic Waves. Risk management and healthcare policy. 2021;14:3775-82. Epub 2021/09/23. doi: 10.2147/rmhp.S326051. PubMed PMID: 34548826; PubMed Central PMCID: PMCPMC8448159.

5. World Health Organization. Contact tracing in the context of COVID-19 Geneva, Switzerland: WHO; 2021 [cited 2022 21 Jan 2022]. Available from: https://www.who.int/publications/i/item/contact-tracing-in-the-context-of-covid-19.

6. Boum Y, Eyangoh S, Okomo MC. Beyond COVID-19-will self-sampling and testing become the norm? The Lancet Infectious diseases. 2021;21(9):1194-5. Epub 2021/04/16. doi: 10.1016/s1473-3099(21)00197-3. PubMed PMID: 33857408; PubMed Central PMCID: PMCPMC8041358.

7. Drain PK. Rapid Diagnostic Testing for SARS-CoV-2. The New England journal of medicine. 2022;386(3):264-72. Epub 2022/01/08. doi: 10.1056/NEJMcp2117115. PubMed PMID: 34995029.

8. World Health Organization. Use of SARS-CoV-2 antigen-detection rapid diagnostic tests for COVID-19 self-testing. Interim guidance. Geneva, Switzerland: WHO, 2022.

9. Indravudh PP, Choko AT, Corbett EL. Scaling up HIV self-testing in sub-Saharan Africa: a review of technology, policy and evidence. Current opinion in infectious diseases. 2018;31(1):14-24. Epub 2017/12/13. doi: 10.1097/qco.0000000000000426. PubMed PMID: 29232277; PubMed Central PMCID: PMCPMC5768229.

10. Choko AT, Desmond N, Webb EL, Chavula K, Napierala-Mavedzenge S, Gaydos CA, et al. The uptake and accuracy of oral kits for HIV self-testing in high HIV prevalence setting: a cross-sectional feasibility study in Blantyre, Malawi. PLoS medicine. 2011;8(10):e1001102. doi: 10.1371/journal.pmed.1001102. PubMed PMID: 21990966; PubMed Central PMCID: PMC3186813.

11. Johnson CC, Kennedy C, Fonner V, Siegfried N, Figueroa C, Dalal S, et al. Examining the effects of HIV self-testing compared to standard HIV testing services: a systematic review and meta-analysis. Journal of the International AIDS Society. 2017;20(1):21594. doi:

10.7448/IAS.20.1.21594. PubMed PMID: 28530049; PubMed Central PMCID: PMCPMC5515051.

12. Stohr J, Zwart VF, Goderski G, Meijer A, Nagel-Imming CRS, Kluytmans-van den Bergh MFQ, et al. Self-testing for the detection of SARS-CoV-2 infection with rapid antigen tests for people with suspected COVID-19 in the community. Clinical microbiology and infection : the official publication of the European Society of Clinical Microbiology and Infectious Diseases. 2021. Epub 2021/08/08. doi: 10.1016/j.cmi.2021.07.039. PubMed PMID: 34363945; PubMed Central PMCID: PMCPMC8336990.

13. Lindner AK, Nikolai O, Rohardt C, Kausch F, Wintel M, Gertler M, et al. Diagnostic accuracy and feasibility of patient self-testing with a SARS-CoV-2 antigen-detecting rapid test. Journal of clinical virology : the official publication of the Pan American Society for Clinical Virology. 2021;141:104874. Epub 2021/06/19. doi: 10.1016/j.jcv.2021.104874. PubMed PMID: 34144452; PubMed Central PMCID: PMCPMC8163726.

14. NHS; UK. Your step-by-step guide to DHSC COVID-19 Self-Test (Rapid Antigen Test). NHS Test and Trace: NHS, 2022.

15. Lindner AK, Nikolai O, Rohardt C, Burock S, Hülso C, Bölke A, et al. Head-to-head comparison of SARS-CoV-2 antigen-detecting rapid test with professional-collected nasal versus nasopharyngeal swab. The European respiratory journal. 2021;57(5). Epub 2021/02/13. doi: 10.1183/13993003.04430-2020. PubMed PMID: 33574072; PubMed Central PMCID: PMCPMC7877326.

16. Simwinga M, Kumwenda MK, Dacombe RJ, Kayira L, Muzumara A, Johnson CC, et al. Ability to understand and correctly follow HIV self-test kit instructions for use: applying the cognitive interview technique in Malawi and Zambia. Journal of the International AIDS Society. 2019;22 Suppl 1(Suppl Suppl 1):e25253. Epub 2019/03/26. doi: 10.1002/jia2.25253. PubMed PMID: 30907496; PubMed Central PMCID: PMCPMC6432102.

17. R Core Team. R: A language and environment for statistical computing. R Foundation for Statistical Computing. Vienna, Austria: 2022.

18. Wachinger J, Olaru ID, Horner S, Schnitzler P, Heeg K, Denkinger CM. The potential of SARS-CoV-2 antigen-detection tests in the screening of asymptomatic persons. Clinical microbiology and infection : the official publication of the European Society of Clinical Microbiology and Infectious Diseases. 2021;27(11):1700.e1-.e3. Epub 2021/07/30. doi: 10.1016/j.cmi.2021.07.020. PubMed PMID: 34325065; PubMed Central PMCID: PMCPMC8310783.

19. Young BC, Eyre DW, Kendrick S, White C, Smith S, Beveridge G, et al. Daily testing for contacts of individuals with SARS-CoV-2 infection and attendance and SARS-CoV-2 transmission in English secondary schools and colleges: an open-label, cluster-randomised trial. Lancet (London, England). 2021;398(10307):1217-29. Epub 2021/09/18. doi: 10.1016/s0140-6736(21)01908-5. PubMed PMID: 34534517; PubMed Central PMCID: PMCPMC8439620.

20. European Centre for Disease Prevention and ControL. Considerations on the use of self-tests for COVID-19 in the EU/EEA – 17 March 2021. ECDC: Stockholm: 2021.

21. UK Government. Order coronavirus (COVID-19) rapid lateral flow tests https://pubmed.ncbi.nlm.nih.gov/34959134/: https://pubmed.ncbi.nlm.nih.gov/34959134/; 2022 [cited 2022 31 Jan 2022]. Available from: https://pubmed.ncbi.nlm.nih.gov/34959134/.

22. Lindner AK, Nikolai O, Rohardt C, Kausch F, Wintel M, Gertler M, et al. SARS-CoV-2 patient self-testing with an antigen-detecting rapid test: a head-to-head comparison with professional testing. Preprint https://wwwmedrxivorg/content/101101/2021010620249009v1. 2021.

23. Choko AT, MacPherson P, Webb EL, Willey BA, Feasy H, Sambakunsi R, et al. Uptake, Accuracy, Safety, and Linkage into Care over Two Years of Promoting Annual Self-Testing for HIV in Blantyre, Malawi: A Community-Based Prospective Study. PLoS medicine. 2015;12(9):e1001873. doi: 10.1371/journal.pmed.1001873. PubMed PMID: 26348035; PubMed Central PMCID: PMCPMC4562710.

24. Peeling RW, Olliaro PL, Boeras DI, Fongwen N. Scaling up COVID-19 rapid antigen tests: promises and challenges. The Lancet Infectious diseases. 2021;21(9):e290-e5. Epub 2021/02/27. doi: 10.1016/s1473-3099(21)00048-7. PubMed PMID: 33636148; PubMed Central PMCID: PMCPMC7906660.

25. Foundation for Innovative New Diagnostics. Test directory https://www.finddx.org/test-directory/2022 [cited 2022 31 Jan 2022]. Available from: https://www.finddx.org/test-directory/.

26. Altman DG, Bland JM. Diagnostic tests. 1: Sensitivity and specificity. BMJ (Clinical research ed). 1994;308(6943):1552. Epub 1994/06/11. doi: 10.1136/bmj.308.6943.1552. PubMed PMID: 8019315; PubMed Central PMCID: PMCPMC2540489.

27. Pant Pai N, Sharma J, Shivkumar S, Pillay S, Vadnais C, Joseph L, et al. Supervised and unsupervised self-testing for HIV in high- and low-risk populations: a systematic review. PLoS medicine. 2013;10(4):e1001414. doi: 10.1371/journal.pmed.1001414. PubMed PMID: 23565066; PubMed Central PMCID: PMCPMC3614510.

28. Reipold EI, Farahat A, Elbeeh A, Soliman R, Aza EB, Jamil MS, et al. Usability and acceptability of self-testing for hepatitis C virus infection among the general population in the Nile Delta region of Egypt. BMC public health. 2021;21(1):1188. Epub 2021/06/24. doi: 10.1186/s12889-021-11169-x. PubMed PMID: 34158006; PubMed Central PMCID: PMCPMC8218412.

S1 text

1. S1 Checklist. Self-sampling checklist-Standard Q
2. S2 Checklist. Self-sampling checklist-PanBio
3. S3 Checklist. Self-testing checklist-Standard Q
4. S4 Checklist Self-testing checklist-PanBio
5. S5 Table. Interpreting contrived panel results
6. S6 Table. User views on self-sampling and self-testing

Click here to access/download
Supporting Information
S1 Text.docx
